# Affections of the Skin Induced by Cold Weather

**Published:** 1886-02

**Authors:** James Nevins Hyde

**Affiliations:** Professor of Skin and Venereal Diseases, Rush Medical College, Chicago


### On the Affections of the Skin Induced by Temperature Variations in Cold Weather. (Second Paper.) *By* James Nevins Hyde, m. d., *Professor of Skin and Venereal Diseases, Rush Medical College, Chicago.*

In the early part of last year* I had the opportunity of writing and publishing a paper on the general subject suggested by the title given above. It is here purposed to supplement that former communication, by touching upon certain points con-

*Chicago Medical Journal and Examiner, March, 1885.

nected with the same theme, that were then omitted chiefly in consequence of lack of space.

For those who are not familiar with the field then surveyed it may be well to note that the subject was introduced by reference to the great prevalence, in the region known as the Northwest, of a class of cutaneous disorders accompanied by pruritus in various grades, and occurring under the influence of temperature-variations in cold weather. It was shown that, while it was comparatively easy to persuade the victims of these disorders as to the actual cause of their trouble when only exposed portions of the body were affected, as for example, in the case of frostbitten hands and feet, it was on the contrary found to be difficult and almost impossible to compass the same end when the climatic influences were exerted upon those portions of the body covered by the clothing.

It was also pointed out that these climatic influences resulted in the production upon the skin of different individuals, of different symptom pictures; one large class of sufferers experiencing only a cutaneous pruritus (the "pruritus hiemalis", of Duhring; "winter prurigo", of Hutchinson) the sole eruption occurring as the result of the traumatisms of scratchin ; and the characteristics of such pruritic skins whether free from eruption or wounded by traumatism were fully enumerated. The several forms of erythema, eczema, urticaria, and furunculosis, resulting from the same causes, namely, sudden temperature-variations, were also described.

It was further stated that most of those holding erroneous opinions respecting the nature of these disorders, could be divided into two classes, viz., first, those entertaining the view that these disorders were parasitic in character; second, those believing that they were due to certain morbid conditions of the system, best recognized under the popular phrase "impurities of the blood." The arguments against both of these conclusions were somewhat fully set forth, the first doctrine being tested by a detailed account of the several points of resemblance and difference between the winter disorders of the skin and scabies, or "the itch."

Attention was directed also to the fact that the acarius scabiei, though requiring an objective of low power for display of its anatomical features, is visible to the naked eye. As I write these lines, I hold before my eyes, whose myopia is corrected for normal vision, a slip of glass to which is attached the body of an average sized female acarus. With the out-door snow for a back-ground, the minute disk of the parasite is visible at a distance of twenty-five inches from the eye; and a medical friend who chances to enter the room at the moment, succeeds in accomplishing the same feat with ease.

Before pursuing this inquiry further I purpose to exhibit some evidence showing the frequency of the disease in certain of the Northwestern and a few of the Southern States of this country; and also the grounds for believing that the group of disorders which here especially concerns us, is of relative infrequency in other parts of the world.

The following extracts from some of the private letters addressed to the author by medical correspondents since the publication of his first paper on this subject, may serve to illustrate the first part of this proposition:

"————, Ill., March 29th, 1885.

"Scabies has existed in this community for about two years, "and is treated here with very little success. Many patients "have suffered for six months before securing relief. I think "there is no doubt as to the nature of the disease.

"Yours very sincerely,

"————."

In this connection it should be noted that the length of time during which some of these patients suffered from the supposed scabies, is that of the cold season in this part of the country.

"———, Ill., Mar. 27th, 1885.

"I have a case of skin disease of the most perplexing character. The patient, a middle-aged lady, is otherwise in the best of health. Her sole trouble is an intolerable pruritus involving every portion of the body. No eruption can be seen. Brisk friction of the surface produces a deep mottling of the integument, with partial relief of the itching. I have had several similar cases, most of them in men, and the treatment in all has been very unsatisfactory. The little son and daughter of the lady whose case is mentioned above, were the only children found to be affected, of some ten or twelve cases. The mother thinks she caught it from the children. No two of the other cases occurred in one family. All were in excellent health and complained only of the itching. I do not believe that it is contagious.

"Yours truly, "——— ———."

"———, Ill., Jan. 2nd, 1885.

"There is a form of scabies in this community which does not yield to the ordinary treatment by sulphur, etc. Can you kindly suggest anything that is capable of curing the disease?" "——— ———."

"———, Iowa, Oct. 30th, 1885.

"I have suffered myself from a very disagreeable attack of itching of the skin which has lasted for about two weeks, existing particularly upon the arms, body, and the inside of the thighs. It scarcely troubles me in the daytime, but is always much worse at night, after retiring to bed and getting in close proximity to a hot fire. I have treated within the last year between two and three hundred cases of this disease and I believe that most of them have recovered in the course of a month. It seems to prevail here as an epidemic.

"Very respectfully yours,

"——— ———."

To this writer, as indeed to all who have sent communications upon the same theme since my first presentation of this subject, a reprint of that first paper was mailed. In consequence, however, of the large experience reported by this correspondent, a letter of specific inquiry was also sent him which elicited the following answer:

"———, Iowa, Nov. 25th, 1885.

"About three years since and at a time when a large force "of laborers was employed on the railroad here, many of them "were affected with a disease of the skin attacking the chest, "sides of the trunk, and arms, characterized by an intense "pruritus with decided nocturnal aggravation. In about two "weeks, about a dozen of these cases were effectually relieved. "After an absence of two years, I returned one year ago to "this place, and found nearly the whole community affected "with what they called 'the itch.' I have since treated be-"tween two and three hundred cases of it and have usually ob-"tained complete relief in from ten to thirty days. I had pa-"tients from all the surrounding towns, some of whom had "been under treatment for two or three months without re-"sulting good.

"A few days since, a day laborer was found dead in his "shanty in the outskirts of this town. Upon removing his "clothing at a coroner's inquest, the entire body was found "covered with a crust surmounted by scales which were nearly "as thick as sole leather. This man had probably suffered "for over two years and presented a picture of the full evolu-"tion of the disease in an untreated case. I do not like to dis-"agree with your opinions, but I must express my belief that "the disease is highly contagious and that its extension in the "community is due far more to this fact than to any atmos-"pheric influences. There is no doubt that a parasite is the "cause, for I have examined both parasite and ova nearly every "day with a glass. I have had it myself four times in the past "year. Certainly no man can pay more attention to personal "cleanliness than I. Even discarding the flannel undercloth-

" ing that I have always worn, has failed to afford me any re-
" lief whatever. We have had here a few cases of genuine
" scabies but they scarcely ever come under the observation of
" a physician, as sulphur and lard soon put an end to them.

" I am with very high regard, etc.,

"______ ______."

To this note an answer was returned begging for a full description of the parasite and its ova; the power of the glass used for its recognition, and a statement as to the habitat of the parasite, whether it burrowed within the skin, or lived upon its surface, or upon the investing clothing.

To this no answer was returned.

"______, Wis., Oct. 8th, 1885.

" I have a patient 63 years old, in excellent general health,
" who has no eruption upon the skin but who, since the first
" of August, has suffered from an intolerable pruritus, with
" considerable aggravation upon retiring to bed at night. The
" result is an insomnia which is exceedingly distressing. The
" sensation of itching seems to be worse on the forearms.
" Other members of the family are affected with the same
" disease.

" Yours very sincerely,

"______ ______."

"______, Wis., Nov. 5th, 1885.

" I wish to inquire about a form of skin disease to which my
" attention has been frequently called during the past year.
" It rages in the camps of the lumbermen; in one instance
" twenty out of thirty men being affected by it, as also a num-
" ber of families where the husband and children suffered if
" not the wife. I have now a merchant under my charge, aged
" 40, whose 5-year old boy has had it and recovered without
" treatment, the wife escaping though, of course, exposed. The
" itching is simply atrocious. Objectively, papules and some-
" times pustules occur upon the extensor surfaces of the limbs.
" I have never found any eruption upon the inter-digital spaces.
" The papules are smaller and more discrete than those in sca-

"bies, and do not yield to sulphur. The patient now under "treatment has the eruption upon the extensor surfaces of the "forearms, scarcely any on the arms; in and about the ax- "illary region; where the waistband of the trousers comes in "contact with the skin; the popliteal spaces; the extensor "faces of the legs; and a few pustules over the thickened pal- "mar integument near the forearm.

"On the anterior surface of the wrist, and also in the palm "of the hand there are two or three pustules; none in the in- "terdigital space. I don't believe this is scabies. I regarded "it as a form of eczema, and treated it accordingly without "success, until I was forced to admit the contagiousness of the "disease, and to adopt anti-parasitic treatment, which, as a "rule, arrested it.

"Is there now recognized, or has there lately been recog- "nized a new parasite, which could be regarded as capable of "producing this disorder? I notice that a writer has lately "claimed that such is the case, particularly in Ohio and the "Northern and Western States. In the lumber districts it is "commonly called 'camp itch,' and other names as little sig- "nificant of its real nature.

"Yours very truly, "————."

"————, Wis.
Nov. 11th, 1885.

"*My Dear Sir:*—I have read very carefully the reprint of "your late paper on 'Winter Disorders of the Skin,' and "after a careful perusal of the same I am almost persuaded of "the truth of your position. The question of contagiousness "is not yet settled in my own mind. When the disease first "came under my observation I regarded it as contagious, "but afterward, as a general eczema due to temperature "changes, until cases occurred in families, where the father, for "example, was the inmate of a lumber camp, became infected, "and immediately after a visit to his family, whose children "were also attacked. The belief is universal that this disease "is contagious.

"Yours very truly, "————."

The following is an interesting communication, on account of the fact that it comes to us from a point much more to the South than any of those heretofore noted.

"————————, Tex.
Nov. 20th, 1885.

"*My Dear Sir:*—Will you kindly give me your opinion of "the following case: A gentleman, 26 years old, and married, "came to me affected with a peculiar itching of the skin over "the iliac crests, extending directly up to the axillæ, and down "to the flexor surface of the arms; no lesions became appar-"ent over the surface until after the skin was scratched, at "which time small wheals appeared. In about a month the "thighs and legs were also affected, and finally the entire "upper extremities, and the body. No treatment has thus far "proved effectual in relieving the pruritus, which is especially "intense at night, and up to the present date has continued "quite unabated.

"Yours very truly,
"————————."

Upon reading this communication, the reprint, to which reference is made above, was mailed to this gentleman, on the supposition that at that season of the year his patient had been subjected somehow to the same influence as others described in this paper. In response I received a communication from him, stating that the patient had been exposed to the action of severe cold in a trip which he had lately made and that after his return to a warmer climate the disease had entirely disappeared.

"————————, Wis.
Jan. 5th, 1886.

"*My Dear Sir:*—I am constantly seeing patients who apply "to me complaining of a severe itching of the surface of the "body, particularly when they are warm, and lying in bed. "On examination the skin may present some small nodules. "The skin is not swollen or reddened, but feels to the touch,

"and is also subjectively, hot and dry. The patients are very apt "to scratch the skin until pin-head-sized nodules appear upon "its surface, which when torn, are covered with spots of dry "blood at the apex of each nodule. Some patients are affect-"ed only upon the arms and legs. Others have it extensively "over the entire surface of the body. Both the flexor and ex-"tensor faces of the limbs are affected. I have not yet seen "the eruption occurring on the feet, hands, face, or genitalia. "Adults, as well as children, are affected. Almost all are male "patients. Some patients have the affection for several years; "others for only a few months or weeks. The health of none "is impaired in the slightest degree. I am quite uncertain as "to the diagnosis. No treatment seems to give relief. The "population here is largely made up of farmers, who eat a "great deal of fat at the table, and most of whom work very "hard. I shall be obliged if you will give me your opinion "upon this question.

"Yours very truly,

"————."

"————————, ILL.

Dec. 24th, 1885.

"*My Dear Sir:*

"In our vicinity there is a 'lichen,' 'itch,' 'mange,' "'scratches,' or something which affects a large number of "people. It is characterized by itching, which is more in-"tense on going to bed at night. The eruption is of the "papular variety, and situated mostly on the extensor "faces of the forearms, the thighs, the breast, and the abdo-"men. My treatment has been anything but satisfac-"tory. I am a young man just beginning the practice of "medicine, and wish to get all the information possible on "the question. You will very much oblige me by giving "me the diagnosis and treatment of this disease.

"Yours very truly,

"———— ————."

Thus much for observation of this group of diseases occurring more particularly in this country. Turning to other countries we are at once struck with the fact that there is almost absolute silence on the part of writers and observers, respecting the prevalence there of such disorders. My paper published last March awakened some attention in England, France and Germany, as brief abstracts of it appeared in the medical literature of those countries, which contained, for the most part, the essential points it presented.

Glancing somewhat hastily over dermatological literature for the year 1885, I have discovered but few references to the disorders under consideration. In the *Annales de Dermat. et de Syphilig.*, for January 25th, 1885, appears an abstract of a paper by Obersteiner, originally published in the *Wien. Med. Wochenschr.*, No. 16, 1884. In this paper is described a case diagnosticated by the author as one of pruritus hiemalis of Duhring. The mere fact that observation of a single case is recorded, alone suffices to suggest its rarity, even if the author did not himself admit, as he does, that it has not been possible to recognize it more often as occurring in the latitude where he is engaged in practice. Obersteiner's single case was that of a young man, 37 years of age, of vigorous constitution, who regularly, every month of October, suffered from a violent pruritus, greatly aggravated in the course of the ensuing winter and disappearing almost entirely in the spring, usually about the month of March. The maximum intensity of the symptoms was noticed at night. Almost all treatment proved useless. The general health was unimpaired, but the sensibility of the integument was greatly exaggerated during the period of access. He notes two points: First, that variations of temperature must be largely responsible for the disease, even excluding severe cold, since his patient lived in Cairo, a point far to the south of Philadelphia, where Duhring first wrote his paper. Second, that the disease is to be regarded as a neurosis, whatever its cause, in view of the fact that his patient found his symptoms influenced by moral emotions. Obersteiner adds that a large number of physicians are reported to have observed the same disease in Austria, but that he does not regard the condition which they describe as identical with the pruritus hiemalis of Duhring.

Turning to the text books written by foreign authors we find descriptions of the group of disorders under discussion in the highest degree meagre and unsatisfactory. Many such make no note of it whatever; others do little more, as for example, Schwimmer, in Ziemssen's Hand Book of Diseases of the Skin, who devotes a single paragraph to the subject, a brief reference in fact to the existence of such a disease in America.

Interesting verbal communications made on the same subject to the writer, by prominent dermatologists and physicians in this country, indicate, as might be expected on *a priori* grounds, a divergence of opinion. Some of both classes are quite confident that a rather larger percentage of scabies is to be credited to the group of disorders here considered, than is represented by the percentage given in the statistics gathered by the committee of the American Dermatological Association. These statistics, however, continue to bear the same testimony upon the point at issue. In the year ending June 30th, 1884, there were three hundred and thirty-nine cases of scabies reported from the several districts, thirty-three occurring in private, and three hundred and six in public practice. At that time, Boston reported one hundred and seventy-nine cases, the percentage of that disease to all cutaneous diseases coming under the notice of the reporters of that district, being ten per cent.

During the succeeding year ending on the 30th of June, 1885, there were four hundred and forty-two cases of scabies reported from the several districts, of which forty-nine occurred in private, and three hundred and ninety-three in public practice. This year, also, Boston exhibited the largest increase, reporting three more than one-half the entire number of cases of scabies registered in the country, viz., 224. It thus appears that the statement made in my former paper still holds good, namely: that it is the cities on the American sea board which are, as a rule, able to report the largest number of cases of scabies in the country; that the cities of the interior must usually furnish a considerably smaller number; and that the towns and villages of the Northwest and West will always suffer less from scabies, than will any other localities in the United States. The most significant exceptions occur where a larger or smaller number of newly-arrived immigrants have produced by settlement a focus in which also a colony of acari have been imported directly from the old world to the new.

But acari and atmospheric influences aside, is it possible that a parasite, hitherto unrecognized and undescribed, works the extensive mischief to which attention has been directed in these papers? Upon this point it is needful to speak with the just reserve based upon all the possibilities of the future. One of the most distinguished observers in this country lately informed me that he had been in correspondence with a gentleman who seemed to have discovered a parasite as the cause of a curious eruptive disorder, similar in character to those described above, and found particularly in Ohio. While ready, however, to admit the truth of all demonstrable facts, present or future, we are in position to-day, to assert that the disorders we have considered, when tested by the standards according to which all contagious diseases of man and animals are proved to be such, fail of conformity in every particular. All the accumulated facts relative to contagion in the winter diseases of the skin, are explainable by the exposure to the same influences at the same time of large numbers of people in a given community, who, it is to be noted, are simultaneously relieved as well as attacked in atmospheric temperatures above or below a certain point.

As to the title or titles which should be given the disorders described above, it remains only to be said, that they must be for the present designated by descriptive terms merely. They constitute a group of affections, for the most part simple in character, the sole reason for their discussion in group, being an etiological one.

The terms "pruritus hiemalis," and "winter prurigo," unquestionably point to the prominent affection in this class, which, however, as I have endeavored to show, exhibit a very wide range of features from pruritus without eruption, and pruritus accompanied by an eruption solely induced by traumatism to extreme grades of inflammation of the skin, characterized by every one of its inflammatory lesions. The recognition of the causes of a disease does not justify the setting of that disease aside in a special category of its own. Pruritus is pruritus, whether induced by a parasite inhabiting the skin, an indigestible aliment in the stomach, or the action of cold upon the outer surface. If need be, for the purpose of convenience, a term may be added referring to its chief etiological factor, which in the present case is the cold weather of the spring, of the fall, or of the winter; or, as has been shown in a previous paper, the relatively sudden changes of temperature from the higher to the lower degrees or the reverse, in any season of the year.

We now turn to the question of treatment:

A perfect prophylaxis and remedy are naturally secured by residence in a climate not subjected to the rapid changes which are experienced in this latitude. When the disease, in any one of its forms, has been pronounced, a removal to such climate is invariably succeeded by a more or less speedy relief of all the symptoms. In touching upon this subject, it is natural that I should refer chiefly to cases seen in Chicago and its vicinity. Patients resident in what is known as "the Upper Lake Region," who have been able when thus afflicted to spend a few weeks in St. Louis, Philadelphia, New York, Baltimore, or at points equally distant from their homes and in similar directions, have usually reported very marked relief within two weeks, and complete recovery in a somewhat longer period. Nor can it be said that this is a merely temporary benefit, for in several cases patients, who, after such residence in other parts of the country, have returned to that in which they first experienced their disagreeable symptoms, have either not suffered from a relapse, or have had the same symptoms occur in a milder form. In general it may be said that the Eastern, South Eastern, extreme Western, and Southern coast line, with some proximity to the sea even in winter, has offered a climate most propitious for securing the desired end. It cannot be said that such relief can be obtained with any degree of certainty in any one of the States forming the Upper

Lake Region, and this, of course, applies equally to the various springs and health resorts in these same limits. Neither can it be said that the method of exposing the skin to the action of heat and hot water, in the Turkish or Russian baths of the large cities, or of the baths in the various Hot Springs that are accessible in the region here defined, can be relied upon for securing the desired result.

It will always be true, however, that the majority of patients who consult physicians will demand what relief is possible for these ailments while they are attending to their business, in the localities where they reside. The problem in these cases is a very great one; it may in fact be described as the great dermatological problem for solution in this part of the world. At times with great ease and brilliant results, at others in the face of disagreeable drawbacks and relapses, the therapeutical solution is always reached in one way or another by clumsier agencies than those exerted by the sun as it nears the summer solstice.

Whatever be the condition of the skin, the following remarks, in regard to the general management of these cases, may be regarded as important. The patient should wear soft, undyed, and unirritating garments next to the skin. Woolen garments, especially those which are highly colored by aniline dyes, and even undyed woolen garments of every sort, should not be worn. Nor can it be said that silk underwear offers any guarantee against pruritic discomfort. It is preferable to wear soft, white, lisle-thread garments next the skin, and outside of these, as may be required by the temperature of the outer air and the vocation of the patient, warm, woolen, or other clothing, necessary to maintain a comfortable bodily temperature.

Patients in this condition should also avoid all ingesta that are capable either of producing or of aggravating cutaneous rashes or pruritic symptoms. Here may be named, among medicaments, arsenic in every form, the salts of iodine and bromine, senna, quinine, chloral, valerian, glycerine, opium, and all remedies which are popularly supposed to "drive out" eruptions upon the surface of the skin, including, of course, every one of the so-called "blood-purifying" remedies.

Among articles of diet it is scarcely necessary to say that oatmeal, which is such a common food in this country, as also wheaten grits, pease, beans, etc., should be avoided. Buckwheat and other hot cakes, pastry, pickles, vinegar; such uncooked vegetables as celery, cabbage and cold-slaw; several of the preserved fruits, including strawberries, gooseberries, and raspberries; all canned fruits, canned meats, and canned vegetables; mushrooms and spinach; salads of all sorts; nuts, raisins, figs, dates, prunes, olives and the skins of grapes; tea, cocoa, and coffee; cheese and sausage; paté de foie gras, Caviare, and Welsh rarebit, game, and all salted, canned, and preserved meats; shell fish, especially lobsters, crabs, and clams have all produced pruritic affections. Fresh oysters in season, and oyster broths are not, however, as a rule, objectionable. Fried meats, fried fish, and fried vegetables are in general to be avoided. Wine, spirits, and beer are not permissible as beverages.

It is evident to any one who carefully examines this list that articles are left unnamed from which an ample dietary may be selected for even an active worker. Thus, one can permit the use of all animal and vegetable soups and broths, without free fat floating upon the surface; fresh, roasted, boiled, broiled and baked meats; poultry; fresh, broiled, boiled, or roasted fish; fresh, cooked vegetables, when not of the class named above; stale wheat bread, eggs, milk, and cream; fresh butter; olive oil as a dressing, fresh fruits, the simple puddings, such as custard, rice, tapioca, and bread puddings; blanc mange, charlotte russe, ice-cream and omelettes.

Tobacco, both by smoking and chewing, is, in the case of male patients, to be interdicted as liable to sustain, if not induce, the irritability of the nervous system which constitutes such a prominent feature of the diseases considered.

As far as possible the patient should avoid extremes of temperature, more particularly at those periods of the day when the pruritus is at its worst; as for example, when the clothing is removed from the body at night, and also when it is replaced in the morning. Unnecessary exposure in an open carriage, or sleigh, should be avoided as liable to awaken the paroxyms of the disease.

It cannot be said that any remedy can be administered internally, which will specifically "cure". The internal treatment of this group of ailments is to be conducted on the general principles by which the physician is guided in the treatment of other simple disorders. Thus, for example, constipation, which is so frequent in the winter season, on account of the necessary restriction of the diet at that time, may be relieved by one of the simple laxatives, such as the mineral waters, the compound licorice powder, or the compound cathartic pill.

In many cases, when the nervous system is suffering greatly from nocturnal paroxysms of distress, a full dose of quinine at night, even though contra-indicated in most cases, taken on retiring to bed, and followed by a cup of warm milk in the morning before rising, will secure very marked results. Other indications are to be met as presented in the case of individual patients.

The local treatment is a matter of prime importance. In the severest cases it is unquestionably necessary to use the hot bath. While it is understood that in general too much bathing is prejudicial to the management of the disorders under consideration, the hot bath is often needed, especially when the patient presents acute inflammatory lesions, raw, tender and torn papules, and infiltrations on the inside of the thighs, the lower belly, and about the knees and forearms. The bath is best taken at night and without soap. To each quart of hot water a teaspoonful of one of the alkalies is added, such as the bi-carbonate of sodium, or the biborate of sodium, or both. After the bath the skin is to be not rubbed, but patted dry, with warm, freshly laundried towels, and a soothing unguent applied. In all cases where it is possible, it is better to not only anoint the surface of the skin with the salve, but to spread the latter upon strips of muslin, to the thickness of a knife blade, which pieces are smoothly applied to the surface, and lightly bandaged, in situ, with a cheese cloth roller. This of course is more applicable to the extremities than to any other portions of the body ; as for example the thighs and the forearms. Any of the simpler salves may be used for this purpose, but that which can be most trusted to accomplish the result in view, is some modification of the long known and still popular unguentum diachyli albi, of Hebra,* which has been called "the prince of ointments."

---

Olei olivæ (opt) ........................ f℥xv.

Lithargyri (opt)........................ ℥iii et ʒ vi.

Aq. font. ........................ q. s.

Mix the oil with a pint of water ; heat in steam bath to boiling. Sift in the litharge in finely powdered state ; stir continually, and boil till all particles have disappeared. Add water occasionally and stir till cool. Immediately after made, unite with equal parts of vaseline. Otherwise, the ointment must be freshly prepared whenever required.

This salve may be mixed with equal parts of vaseline and cold cream, and to each ounce of the whole may be added from five to fifteen grains of salicylic acid, the whole forming a compound which usually affords a great deal of comfort to the surface.

In place of this, the soothing salves, containing bismuth, creta preparata, and the oxide of zinc may be employed. A very useful formula is the following:

| | |
|---|---|
| Oxide of bismuth | ʒi |
| Oleic acid | ℥i |
| White wax | ʒiii |
| Vaseline | ʒix |
| Oil of roses | ♏ii |

M. S. A. [Anderson.]

In many cases however, the skin is in a state of such high inflammation that a still more soothing method of treatment must be pursued. In these cases it is exceedingly useful to adopt the plan to which resort is often had in the management of acute eczema. A lotion of the kind indicated in the following formula is thoroughly shaken up in its vial, poured into a cup or saucer, and then daubed over the part with a mop made of rags, rather than with a sponge, which only serves to entrap the insoluble portion of the lotion:

| | |
|---|---|
| Creta preparata | ʒi |
| Oxide of zinc | ʒi |
| Dilute hydrocyanic acid | ʒss |
| Glycerine | ℥ss |

Lime water and elder-flower water each Oss.

While this is daubed over the surface with one hand, the other is employed in gently rubbing into the skin one of the soothing ointments already described. By one or more of these means, the patient can be usually relieved to an extent sufficient to ensure a comfortable night. In the morning, one of several courses may be pursued. Either the patient remains in his room, which should be heated to a barely comfortable temperature, ranging from 68° to 72° Fahrenheit, and remains there smeared with his salve dressing, practically as he was during the night; or if he or she must necessarily atttend to the active employment of out-door life, the skin is lightly anointed with one of the salves, and then over it is dusted one of the powders which are used for that purpose. Inasmuch as a large portion of the skin has often to be protected in this way, the powder selected should be exceedingly fine, soft to the touch, and comparatively inexpensive. Few are better than the Oswego Gloss Starch which is sold by the grocer, though the several dusting powders used in medicine may be substituted for it in order to induce a greater anti-pruritic effect; as for example combinations of talc, salicylic acid, camphor, oxide of zinc, starch, Fuller's earth, and lycopodium.

Dr. Faithful, of Australia, has recently suggested a new method of preparing medicated powders for skin affections of this class. The remedy is first dissolved in alcohol, ether, or chloroform; the solution is then mixed with starch, or French chalk, and the menstruum allowed to evaporate. The evaporation is conducted without the aid of heat, and a fine medicated starch or chalk powder remains.

In many cases one or more of the lotions esteemed in the management of pruritus in general can be here also employed with advantage. Some of the formulæ of this class are suggested in the appended note.*

---

* Lotions containing the tars (ol. cadini, ol. fagi, ol. rusci) and carbolic acid, one to two per cent.; naphthol solutions, three to five per cent.; *e. g.*, ℞. ol. rusci ʒ iiss, glycerin., ʒ v, spts. vin. rect., ℥v, lavend. spts., gtts. xx. M.

In the milder forms of this group of affections less elaborate local treatment may be adopted. Thus for example, the bathing may be omitted, and the salve simply applied by inunction of the affected surface of the body, great precaution being taken as to the character of the clothing worn next to the skin. Often it happens that this unirritating material, after having taken up some of the salve applied to the surface, is itself a dressing, which, disregarding for a few days the question of cleanliness, suffices to secure a great deal of relief. It is rare, even in these milder cases, that the dusting powder can be with advantage applied directly to the surface, as its peculiar "drying" effect is irritating to the surface of the skin. Many patients in this condition experience relief from taking any one of the simple salves, such as vaseline, cold cream, mutton tallow, and, as Fox, of New York, has lately suggested, the balsam of fir, lightly anointing the affected surface with it, and over that thickly dusting the powder selected for the individual case.

When the active evidences of the disease cease to appear, the infiltration of the skin, which is frequently left, will as a rule slowly subside under careful hygienic management.

Especial care must be taken, in all cases, of the extremities, more particularly of the hands and the wrists. The gloves worn during the seasons of the year when the temperature variations are most sudden and the mercury approaches the zero point, should be preferably of kid,

---

(Schwimmer.) ℞. Acid. salicylic., ʒ i, spts. vin. rect. ℥ii aq. dest., Oiss M. Solutions of corrosive sublimate 0.008 to 200. of water (Trousseau). ℞. Thymol. grs. xv. glycerin., spts. vin. rect., aa ʒ v, aq. dest. Oiss M. Plumbi acet., grs. xx to ℥ii of water; chloral-camphor, 1 part; glycerine, 2 parts; distilled water, fifty parts (some camphor will be here precipitated). ℞. Boracis, ʒ ii, glycerin., ℥i, spts. camph. ℥ss. aq. ros. ℥viss (Duhring). ℞. Glycerit. plumb. acetat. ʒ i, glycerin. ʒ ii, aq. ros. ad ℥iv. M. (Squire).

covered with the muff or with the fur glove. Woolen gloves and those lined with lambs' wool, flannel, and fur, are particularly harmful.

Attention was called in my first paper to the effects produced at the wrist by the play of the cuff over the surface, both the linen cuff and the edge of the sleeve of the coat, "waist," great-coat, or cloak. These parts are best protected by strips of cloth, spread with the salve, and neatly fastened over the surface from a point extending beyond the bases of the metacarpal bones, to the middle of the forearm. The back and sides of the neck when affected should also be protected from the linen collar and ruching, as also from the collar of the cloak, coat, and waist, by a silk handkerchief.

Latapin*, in the Proceedings of the Caucasian Medical Society, has lately advised for frost-bitten fingers and toes the painting of the surface with a mixture of dilute nitric acid, and peppermint water in equal proportions. After this application has been made for three or four days, the skin becomes dark, and the epidermis is shed, a healthy skin appearing underneath. He has found this plan very effectual among soldiers, who were unable to wear their boots, and in consequence have had frozen feet.

Few of the more modern devices of the therapeutist in dermatology are as useful in the management of these disorders as in others. The salben-mülle and pflaster-mülle, that come to us from abroad, are more applicable to cutaneous disorders of limited area. The salve-muslins, however, containing salicylic acid, thymol, zincoxyd, carbolic acid, and ichthyol, may be used over the fingers, toes, wrists,

* Brit. Med. Jour., Sept. 5, 1885.

forearms, and ankles; also, in the way suggested by my friend, Dr. Hardaway, of St. Louis, as linings for the hat, cuffs, and collar.

